# Choroidal thickness in patients with thyroid-associated ophthalmopathy, as determined by swept-source optical coherence tomography

**DOI:** 10.1136/bjo-2023-323694

**Published:** 2023-10-19

**Authors:** Sisi Zhong, Fanglin He, Sijie Fang, Jing Sun, Yinwei Li, Zhang Shuo, Xingtong Liu, Xuefei Song, Yang Wang, Yazhuo Huang, Huifang Zhou, Xianqun Fan

**Affiliations:** 1 Department of Ophthalmology, Shanghai Ninth People's Hospital, Shanghai JiaoTong University School of Medicine, Shanghai, China; 2 Shanghai Key Laboratory of Orbital Diseases and Ocular Oncology, Shanghai Ninth People's Hospital, Shanghai JiaoTong University School of Medicine, Shanghai, China; 3 Shanghai Institute of Immunology, Shanghai JiaoTong University School of Medicine, Shanghai, China; 4 Department of Immunology and Microbiology, Shanghai JiaoTong University School of Medicine, Shanghai, China

**Keywords:** Orbit, Inflammation, Choroid, Imaging, Diagnostic tests/Investigation

## Abstract

**Aim:**

This study used swept-source optical coherence tomography (SS-OCT) to investigate subfoveal choroidal thickness (SFCT) in patients with thyroid-associated ophthalmopathy (TAO) who displayed different levels of disease activity and severity.

**Methods:**

Thirty patients with TAO (60 eyes) and 38 healthy controls (67 eyes) in Shanghai, China, were recruited for this study. Disease activity and severity were graded using European Group on Graves’ Orbitopathy standardised criteria. SFCT values were determined by SS-OCT.

**Results:**

In total, 129 eyes were included in the final analysis. The mean SFCT was significantly thicker among patients with active disease (276.23±84.01 µm) than among patients with inactive disease (224.68±111.61 µm; p=0.049) or healthy controls (223.56±78.69 µm; p=0.01). There were no differences in SFCT among patients with moderate-to-severe disease, patients with severe disease and healthy controls (p>0.05). Changes in SFCT demonstrated strong predictive ability to distinguish active TAO from inactive TAO (area under the curve=0.659, 95% CI 0.496 to 0.822).

**Conclusions:**

SFCT was strongly associated with Clinical Activity Score in patients with TAO. Choroidal thickening was observed during active TAO. SS-OCT offers a non-invasive method for follow-up assessment.

**WHAT IS ALREADY KNOWN ON THIS TOPIC**

- The staging of thyroid associated ophthalmopathy depends on the evaluation of clinical symptoms presently. However, individual differences in clinical manifestations are significant, and the evaluation is based on the subjective experience of clinical doctors.

**WHAT THIS STUDY ADDS**

- This study aims to find objective indicators to assist in disease staging. We measured the choroidal thickness of thyroid-associated ophthalmopathy (TAO) patients in different stages using swept-source optical coherence tomography (SS-OCT) and found that the choroidal thickness of TAO patients in the active phase increased significantly. The accuracy of activity prediction using subfoveal choroidal thickness is high.

**HOW THIS STUDY MIGHT AFFECT RESEARCH, PRACTICE OR POLICY**

- SS-OCT is a useful and non-invasive tool in orbital disease research. We could use optical examination to assist the staging of TAO and further provide evidence for clinical decision.

## Introduction

Thyroid-associated ophthalmopathy (TAO) is the most common extrathyroidal complication in patients with the autoimmune condition Graves’ disease.^1^ After an initial inflammatory (active) phase and a stabilisation (plateau) phase, TAO tends to improve and eventually becomes inactive (also known as burnout phase).^2^ Patients can present with mild, moderate-to-severe or sight-threatening symptoms.^3^ Accurate clinical assessment of TAO, including grading of disease activity and severity, is necessary for early diagnosis, identification of patients likely to develop more serious complications, and appropriate clinical management.^4 5^ Evaluation of TAO activity and severity is based on multiple clinical features that may be perceived differently by various physicians. Therefore, a more objective evaluation method is needed for consistent early diagnosis.

The active phase of TAO is characterised by periorbital oedema and congestion, which ultimately result in orbital fibrosis, glycosaminoglycan deposition and enlarged extraocular muscles.^6^ The choroid is the posterior part of the uvea (ie, the vascular tunic) and mostly consists of blood vessels. In recent years, retinal thickness overall and choroidal thickness in particular have been regarded as potential markers of structural inflammation. Changes in choroidal thickness can be observed in various inflammatory diseases (eg, Behçet’s disease).^7 8^ Choroidal thickness may be a useful parameter for disease monitoring and prognosis prediction. Swept-source optical coherence tomography (SS-OCT) is the latest advancement in retinal and choroidal imaging; it enables real time, in situ acquisition of high-resolution, non-invasive cross-sectional subsurface tomographic images depicting biological structures.^9^ The emergence of SS-OCT offers an improved method for measurement of choroidal thickness. Its 1050 nm wavelength is superior to the 840 nm wavelength used in spectral domain OCT (SD-OCT).^10^ SS-OCT can overcome ocular opacities (eg, cataracts and vitritis), revealing the retina and choroid in eyes where the fundus is not fully visible.

In this descriptive cross-sectional study, we used SS-OCT to investigate choroidal thickness in patients with various levels of TAO activity and severity. We sought to identify an objective parameter that could be used as a marker for assessment of disease activity.

## Materials and methods

### Study population and design

This descriptive cross-sectional study was performed at the Department of Ophthalmology in Shanghai Jiao Tong University School of Medicine Affiliated Ninth People’s Hospital between December 2021 and May 2022. The principles outlined in the Declaration of Helsinki were followed.

Thirty patients with TAO (study group) and 38 age-matched and sex-matched healthy controls (control group) were included in the study. One volunteer in the control group had only one eye included, due to high myopia in single eye, which could have affected the measurement of choroidal thickness. The images of eight eyes in the control group were unclear because of refractive medium opacity or poor cooperation. Thus, 67 eyes were included from the 38 healthy controls. The study group comprised patients with TAO who had been referred to this study by clinicians in the Department of Ophthalmology. All patients were diagnosed with TAO using European Group on Graves’ Orbitopathy (EUGOGO) criteria. The control group comprised healthy individuals without vitreoretinal diseases (except the excluded eyes mentioned above) who had been examined in the Department of Ophthalmology.

The exclusion criteria for this study were prior history of significant ocular disease; amblyopia; refractive error of less than −3 dioptres (D) or more than +3 D; intraocular pressure (IOP) readings greater than 21 mm Hg; glaucoma; history of uveitis, retinal disease, ocular trauma or tumour; poor image quality; pregnancy; dense media opacity; and any associated systematic disorders that could affect the eyes (eg, uncontrolled diabetes, systemic corticosteroids or hypertension).

### Examination protocol and study measurements

Detailed history, subjective symptoms, symptom duration and duration of thyroid disease were recorded by a single ophthalmologist at the first visit. History of smoking, hyperthyroidism treatment and family history were also recorded. The Clinical Activity Score (CAS) was used to grade disease activity. Disease severity was assessed using standardised criteria proposed by the European Thyroid Association/EUGOGO.^4 11^

All patients underwent standard ophthalmic examinations, including Snellen best-corrected visual acuity (BCVA) and refraction, IOP measured by non-contact tono-pachymetry and exophthalmometry using a Hertel exophthalmometer. Axial length was measured using a partial coherence laser interferometer (Zeiss IOL Master; Carl Zeiss AG, Oberkochen, Germany). Colour vision was evaluated using the Ishihara test. Diplopia and eye movement were examined in the nine cardinal positions of gaze. After ophthalmic examinations, all participants were assessed by SS-OCT (three-dimensional deep range imaging OCT Triton (Plus), Topcon Corporation, Tokyo, Japan). All ophthalmic examinations were performed between 10:00 and 12:00 hours.

### SS-OCT measurement

**Figure 1:**
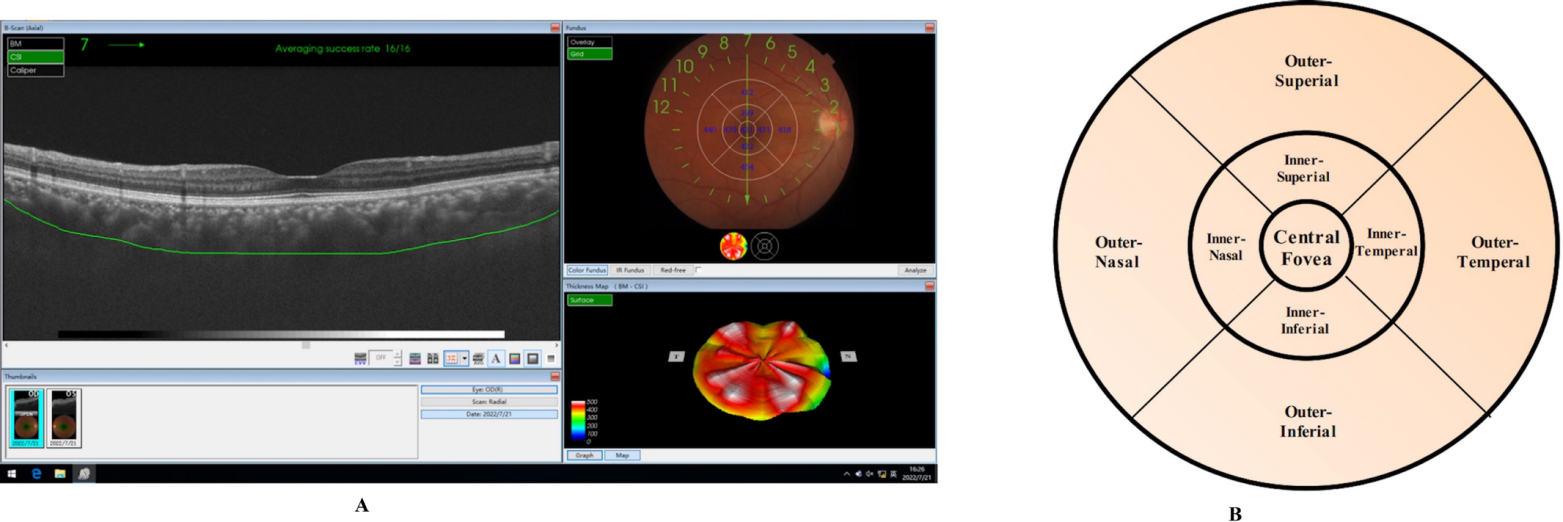
(A) Imaging of the retinal and automatic recognition of the choroidal boundary using SS OCT. Choroid thickness values in nine zones can be obtained. Thickness map can be rebuilt after data acquisition. (B) The standard ETDRS grid demonstrating superior, inferior, temporal and nasal quadrants zone of both inner and outer ring. ETDRS, early treatment diabetic retinopathy study; SS-OCT, swept-source optical coherence tomography.

Choroidal thickness was measured by SS-OCT (figure 1A). The standard ETDRS grid has inner and outer rings, with respective diameters of 1–3 and 3–6 mm; these rings are divided into superior, inferior, temporal and nasal quadrants (figure 1B). The thickness values are mean values calculated in each sector of the grid. Choroidal thickness was measured as the distance between Bruch’s membrane and the choroid-sclera interface. Retinal thickness was measured as the distance between the internal limiting membrane and the interface between photoreceptor outer segments and retinal pigment epithelium. Ganglion cell layer (GCL) thickness was measured as the distance from the interface between the retinal nerve fibre layer (RNFL) and GCL to the interface between the inner plexiform layer (IPL) and inner nuclear layer. GCL+ was regarded as the cumulative thickness of IPL and GCL. GCL++ was regarded as the cumulative thickness of IPL, GCL and RNFL. RNFL thickness was measured as the distance between the internal limiting membrane and the interface between the RNFL and GCL.

### Statistical analyses

Data were analysed using SPSS Statistics, V.22.0 (IBM). Quantitative data are presented as means±SD. Normality was assessed using the Kolmogorov-Smirnov test (p>0.05). Statistical differences in variables that followed a normal distribution were evaluated using independent samples t-tests (two groups) or analysis of variance (≥3 groups). Categorical variables were compared using the χ^2^ test. A p<0.05 was regarded as the threshold for statistical significance. Multivariate linear regression was used to determine the quantitative relationship between more than two dependent variables.

## Results

This study included 30 patients with TAO (60 eyes) and 40 age-matched and sex-matched healthy controls (67 eyes). Demographic data and clinical characteristics are shown in online supplemental etable 1. The two groups did not significantly differ in terms of axil length or BCVA (p=0.5865, p=0.6187).

10.1136/bjo-2023-323694.supp2 Supplementary data

Patients were divided into active and inactive groups according to their CAS; healthy individuals were regarded as the control group. Subfoveal choroidal thickness (SFCT) values were calculated and compared among active disease, inactive disease and control groups. The results are shown in table 1 and online supplemental efigure 1. The mean SFCT in patients with active disease was 276.23±84.01 µm, whereas it was 224.68±111.61 µm in patients with inactive disease. The mean SFCT in all patients with TAO was 257.33±97.40 µm, whereas the mean SFCT in healthy controls was 223.56±78.69 µm. The Kruskal-Wallis test showed that differences among the groups were statistically significant (p=0.005). Dunn’s test, using the Bonferroni method to correct for multiple comparisons, showed that the mean SFCT was significantly thicker in the active group than in the inactive group (p=0.049); the mean SFCT was also significantly thicker in the active group than in the control group (p=0.010). Furthermore, the difference in mean SFCT between the TAO and control groups was statistically significant (p=0.03). The mean SFCT was thicker in the control group than in the inactive group, but the difference was not statistically significant (p>0.05).

**Table 1:** SFCT values of patients in active phase, inactive phase and healthy controls

Group	N	Mean	Min	Max	SD		P value
Active group (group 1)	38	276.23	113.29	483.86	84.01	Group 1 vs group 2	0.049
Inactive group (group 2)	22	224.68	63.67	410.91	111.61	Group 1 vs group 3	0.010
TAO (group 1+2)	60	257.33	63.67	483.86	97.40	TAO vs normal control	0.03
NC (group 3)	67	223.56	61.63	415.98	78.69	Group 2 vs Group 3	1.000

NC, normal control; SFCT, subfoveal choroidal thickness; TAO, thyroid-associated ophthalmopathy.

Patients were divided into mild, moderate-to-severe and severe groups according to disease severity; these groups were compared with the control group described above. The results are shown in table 2 and online supplemental efigure 1. Tukey’s honestly significant difference test, using the Bonferroni method to correct for multiple comparisons, showed that the mean SFCT tended to be thicker in the severe group than in the moderate-to-severe group (mean difference: 2.38, range: −65.322 to 70.082, p>0.05). The mean SFCT tended to be thinner in the control group than in the moderate-to-severe group (mean difference: −35.9, range: −75.327 to 3.526, p>0.05). The mean SFCT tended to be thinner in the control group than in the severe group (mean difference: −38.28, range: −103.89 to 27.329, p>0.05).

**Table 2:** SFCT values of moderate to severe, severe TAO patients and healthy controls

Group	N	Mean	Min	Max	SD		P value
Moderate to severe group (group 1)	48	256.85	63.67	450.97	100.14	Group one vs Group 2	0.996
Severe group (group 2)	12	259.23	158.72	483.86	89.61	Group one vs Group 3	0.118
NC (group 3)	67	223.56	61.63	415.98	78.69	Group two vs Group 3	0.405

SFCT, subfoveal choroidal thickness; TATO, thyroid-associated ophthalmopathy.

Next, we investigated the thicknesses of the five layers in each quadrant, as shown in table 3. We found that only choroid (not the remaining four layers) is affected in TAO. Although choroidal thickness varied among regions, we found that the choroid was thicker in the active group (compared with the inactive group) in the central, inner inferial, inner nasal, outer nasal regions (p<0.05) and outer inferial (p<0.01).The difference was more prominent between the active and control groups in central, inner inferial, outer inferial (p<0.01), inner nasal and outer nasal (p<0.05) regions. Among the nine early treatment diabetic retinopathy study (ETDRS) regions, choroidal thickness in the active group was thinnest in the nasal outer macula (215±79 µm), whereas it was thickest in the inner temporal macula (284±88 µm).

**Table 3:** Topographic characteristics of choroid and retina in TAO patients and healthy controls

9 Zones	5 Layers	Active (n=38)		Inactive (n=22)		NC (n=67)		Total (n=127)		P active versus inactive	P active versus NC	P inactive versus NC
Sub field	Thickness	Mean	SD	Mean	SD	Mean	SD	Mean	SD			
Central fovea	Choroid	276	84	225	112	224	79	240	89	<0.05*	＜0.01**	1.00
	Retina	254	60	255	23	250	44	252	46	0.97	0.96	0.17
	GCL+	53	14	55	12	53	18	53	16	1.00	1.00	0.42
	GCL++	66	31	64	19	65	36	65	32	1.00	1.00	0.75
	RNFL	13	20	10	8	12	20	12	18	1.00	1.00	1.00
Inner temperal	Choroid	284	88	230	106	89	9	248	88	0.07	＜0.05*	1.00
	Retina	297	59	296	17	295	25	296	38	0.37	1.00	0.87
	GCL+	82	13	85	12	83	12	83	12	0.46	1.00	1.00
	GCL++	109	20	107	14	109	15	108	17	1.00	1.00	1.00
	RNFL	27	14	22	5	26	15	26	14	1.00	1.00	1.00
Inner superial	Choroid	275	77	229	109	60	10	247	85	0.06	0.07	1.00
	Retina	307	33	313	19	313	45	311	38	0.14	0.88	0.59
	GCL+	87	8	87	11	87	11	87	10	1.00	1.00	1.00
	GCL++	120	12	124	14	126	29	124	23	0.25	0.41	1.00
	RNFL	33	9	37	9	39	24	37	19	0.16	0.28	1.00
Inner nasal	Choroid	257	83	203	114	205	85	220	93	＜0.05*	＜0.05*	1.00
	Retina	307	28	313	21	310	23	310	24	0.17	1.00	0.59
	GCL+	87	10	89	13	89	9	89	10	0.38	0.55	1.00
	GCL++	118	14	119	17	120	14	119	14	0.30	0.22	1.00
	RNFL	31	13	30	10	31	13	31	12	1.00	1.00	1.00
Inner inferial	Choroid	272	85	219	120	218	82	235	93	＜0.05*	＜0.01**	1.00
	Retina	295	31	301	16	300	16	298	22	0.58	0.37	1.00
	GCL+	78	16	86	11	85	10	83	13	0.13	0.07	1.00
	GCL++	111	18	112	17	117	15	114	16	1.00	0.27	1.00
	RNFL	33	19	26	8	31	12	31	14	0.47	1.00	0.18
Outer temperal	Choroid	279	83	230	92	234	71	247	81	0.07	＜0.05*	1.00
	Retina	260	41	261	18	254	18	257	27	1.00	1.00	0.58
	GCL+	68	10	69	8	66	9	67	9	1.00	1.00	0.88
	GCL++	96	14	95	11	93	10	94	11	1.00	1.00	1.00
	RNFL	28	9	26	6	27	9	27	9	0.95	0.50	1.00
Outer superial	Choroid	268	72	225	96	242	71	247	77	0.11	0.38	0.91
	Retina	275	41	270	16	275	49	274	43	1.00	1.00	1.00
	GCL+	64	10	59	8	61	10	62	10	0.53	1.00	1.00
	GCL++	109	15	107	12	113	28	111	23	1.00	0.62	0.28
	RNFL	45	10	47	9	51	22	49	18	0.95	＜0.05*	1.00
Outer nasal	Choroid	215	79	168	116	167	86	182	92	＜0.05*	＜0.05*	1.00
	Retina	286	24	285	16	285	18	285	19	1.00	1.00	1.00
	GCL+	68	9	65	13	66	9	66	9	1.00	0.78	1.00
	GCL++	123	13	121	13	124	11	123	12	1.00	1.00	1.00
	RNFL	55	14	56	15	59	12	57	13	1.00	0.11	0.67
Outer inferial	Choroid	264	80	205	114	210	80	225	90	＜0.01**	＜0.01**	1.00
	Retina	264	27	265	19	259	17	262	21	1.00	0.53	0.52
	GCL+	60	13	63	10	60	10	60	11	1.00	1.00	0.92
	GCL++	108	12	108	12	107	10	107	11	1.00	1.00	1.00
	RNFL	48	12	46	10	47	9	47	10	0.60	1.00	0.65
Average thickness	Choroid	261	76	210	103	216	74	228	82	＜0.05*	＜0.05*	1.00
	Retina	278	31	277	14	276	21	277	23	1.00	1.00	1.00
	GCL+	69	6	69	7	68	6	68	6	1.00	1.00	1.00
	GCL++	109	11	108	11	110	11	109	11	1.00	1.00	1.00
	RNFL	40	9	40	8	42	10	41	9	1.00	0.57	0.31

*p<0.05，**p<0.01, ***p<0.001

GCL, Ganglion cell layer; RNFL, retinal nerve fiber layer; TAO, thyroid-associated ophthalmopathy.

**Figure 2:**
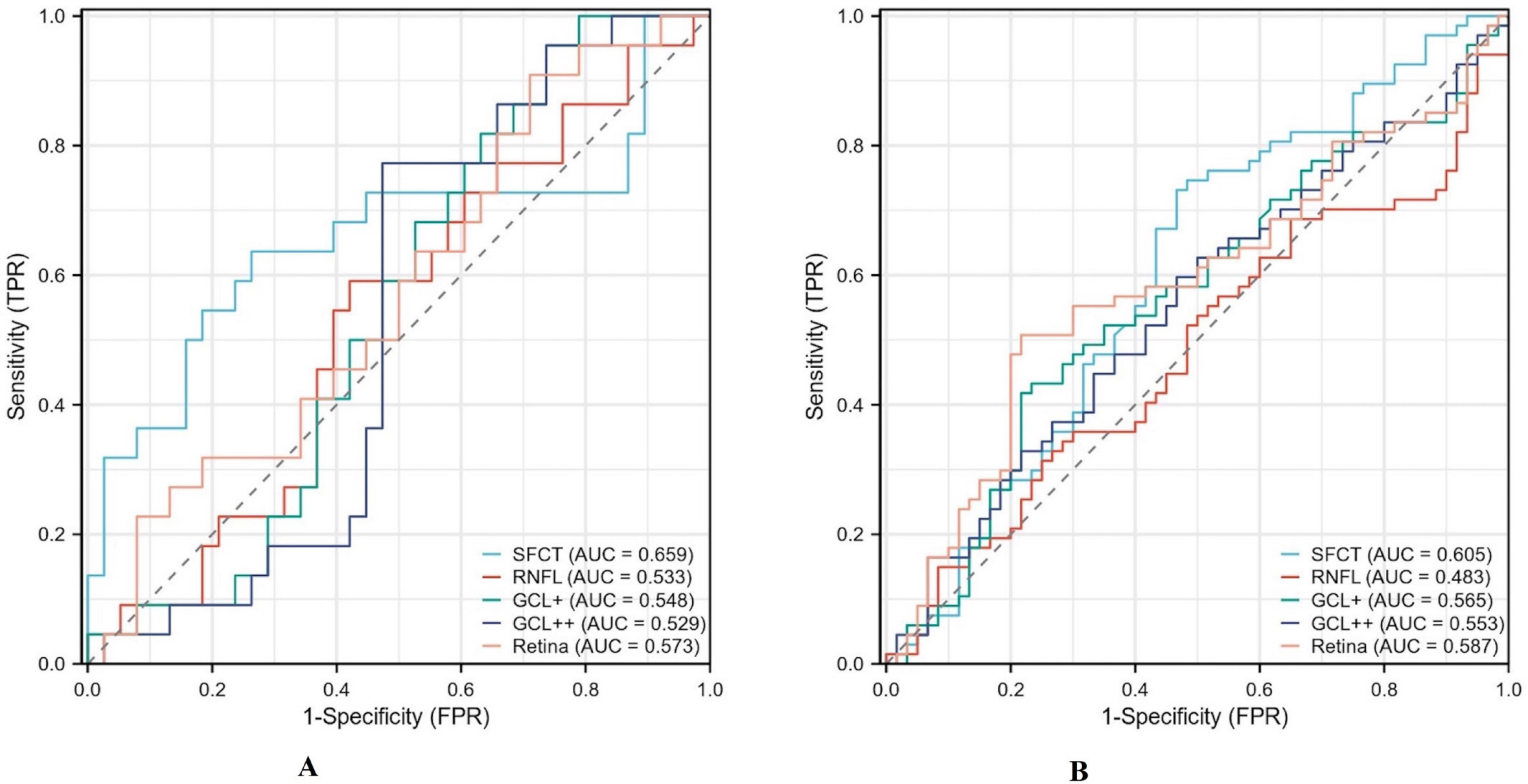
(A) ROC curve of SFCT, RNFL, GCL+, GCL++ and retina in the diagnosis of active TAO. (B) ROC curve of SFCT, RNFL, GCL+, GCL++ and retina in the diagnosis of TAO. AUC, area under the curve; FPR, false positive rate; GCL, ganglion cell layer; ROC, receiver operating characteristic; SFCT, subfoveal choroidal thickness; TAO, thyroid-associated ophthalmopathy.

SFCT demonstrated strong predictive ability to distinguish active TAO from inactive TAO (area under the curve (AUC)=0.659, 95% CI 0.496 to 0.822); RNFL, GCL+, GCL++ and retinal thicknesses had weak predictive abilities (AUC=0.533, 0.548, 0.529 and 0.573) (figure 2A). We compared receiver operating characteristic curves to determine which of these five variables could clearly distinguish the TAO group from the control group. As shown in figure 2B, SFCT (AUC=0.605) had a better diagnostic effect than the other four variables.

We hope to find out the relevant factors that affect the choroid thickness. Multivariate analysis of the TAO group was performed with SFCT as the dependent factor; age, duration of disease, proptosis value, CAS, BCVA, IOP and axial length were the independent factors (online supplemental etable 2). SFCT displayed the following associations: negative (β=−43.34) with axial length (p<0.001), positive (β=8.28) with IOP (p<0.001), positive (β=105.11) with BCVA (p=0.005) and positive (β=21.68) with CAS (p=0.019). These results indicate that choroidal thickness decreases as axial length increases; conversely, choroidal thickness increases as CAS and IOP increase.

## Discussion

TAO is an autoimmune inflammatory disease in which orbital tissues undergo extensive remodelling; this constitutes a challenging ocular component of Graves’ disease. The global prevalence is approximately 0.1%–0.3%.^12^ The most common clinical features of TAO are exophthalmos, upper eyelid retraction, restrictive strabismus, diplopia and optic neuropathy. Approximately 3%–5% of patients with TAO have severe disease involving intense pain, inflammation and sight-threatening corneal ulceration or compressive optic neuropathy. The main processes involved in TAO pathogenesis are cytokine production and inflammation, which can lead to orbital adipose tissue expansion and extraocular muscle oedema within the orbit. Early intervention during active disease can prevent TAO from worsening to dysthyroid optic neuropathy (DON).

Clinically, the selection of a treatment regimen depends on the assessment of disease activity and severity. The current staging method is based on CAS, which mainly involves seven clinical manifestations: spontaneous retrobulbar pain, eye rotation pain, eyelid oedema, eyelid congestion, conjunctival oedema, conjunctival congestion and lacrimal caruncle swelling. TAO is defined as active if the score is ≥3/7. However, patients’ symptoms tend to fluctuate and exhibit large individual differences; the lack of objective and quantitative evaluation criteria leads to subjective judgments.

The choroid mostly consists of blood vessels that supply oxygen to the outer retina, retinal pigment epithelium and possibly the prelaminar portion of the optic nerve.^13^ Choroidal thickness is an objective parameter that describes choroidal morphology and function.^14^ Some inflammatory disorders (eg, Vogt-Koyanagi-Harada disease and Behçet’s disease) affect the choroid during the acute stage of disease.^15 16^ However, the measurement of choroidal thickness has remained a challenging problem.

A key component of choroidal thickness measurement involves identifying the boundary between the choroid and sclera. Previous techniques, such as colour Doppler flow imaging, were either non-quantitative or invasive. SD-OCT has been used for quantitative assessment of the retina.^10^ Çalışkan *et al* used SD-OCT to demonstrate that the mean SFCT was increased in patients with active TAO.^17^ However, SD-OCT requires manual measurement, which leads to poor homogeneity. SS-OCT is a newer technology that can be used to measure choroidal thickness.^9 18^ Compared with SD-OCT, the longer wavelength of SS-OCT can overcome ocular opacities such as cataracts and vitritis, allowing visualisation of the retina and choroid in eyes where the fundus is not fully visible. Moreover, boundaries are automatically and clearly identified, thus providing an important technological advancement in the measurement of choroidal thickness.

Previous studies using SD-OCT showed that the choroid was significantly thicker in the eyes of patients with active TAO.^19^ Consistent with previous reports, we found patients with active TAO had greater choroidal thickness. Patients with active disease had significantly greater choroidal thickness values, compared with inactive patients and healthy controls. Notably, we found a significant association between SFCT and disease activity, where SFCT was significantly thicker during active disease than during inactive disease; this finding suggests that SFCT can serve as an auxiliary marker of disease activity. Receiver operating characteristic curve analysis indicated that SFCT was superior to retinal, RNFL, GCL, GCL+ and GCL++ thicknesses in terms of judging disease activity.

Choroidal thickening may be explained by superior orbital vein (SOV) congestion.^20^ There is evidence that SOV flow is reduced during active disease.^21^ Konuk *et al* found that SOV blood flow velocity was significantly lower in patients with severe TAO than in patients with moderate TAO, suggesting that SOV may influence the clinical course of DON.^22^ Nik *et al* found that patients with Graves’ orbitopathy may have abnormal choroidal perfusion, even in the absence of optic neuropathy. Orbital decompression can improve choroidal circulation in these patients.^23^ Therefore, a decrease in orbital venous drainage may be the cause of increased SFCT in patients with active TAO.

Active disease is characterised by orbital adipose tissue expansion and extraocular muscle oedema within the orbit. The overexpression of thyrotropin receptor and insulin-like growth factor-1 receptor triggers autoimmune reactions, which lead to inflammatory infiltration and glycosaminoglycan accumulation.^24 25^ The accumulation of hyaluronan acid and collagen contributes to greater osmotic pressure and draws water into retrobulbar tissue, potentially resulting in choroidal oedema and thickening. An interesting finding in our research is that the choridal thickens significantly in inferial and nasal region. Traditionally, extraocular muscles of TAO patients are said to be involved in the following order, inferior rectus, medial rectus, superior rectus and lateral rectus.^26^ The consistency between them provides clues to identify the potential causes of choroidal thickening.

Choroidal thickness is also affected by other factors, including sex, age, dioptre and axis length. We found that choroidal thickening in patients with active TAO was associated with short ocular axis, higher CAS, higher IOP and better visual acuity. There were no relationships with age, proptosis or duration of disease. Ding *et al* found that the mean SFCT among individuals aged <60 years was 294.63±75.90 µm, but they did not report an association between SFCT and age.^27^ A relationship between axial length and choroidal thickness has been reported in many studies. Prousali *et al* showed that myopic eyes had thinner choroidal tissue, compared with emmetropic and hyperopic eyes.^28^ Tian *et al* found that a thinner temporal choroid at age 12 was associated with greater 1-year axial elongation in students with myopia.^29^ Xie *et al* demonstrated that choroidal thickness was negatively associated with age among individuals aged >50 years. As axial length increased, choroidal thinning was more prominent in central and parafoveal regions.^30^ All of the above findings suggest a negative association between choroidal thickness and axial length. The results of this study are similar to the findings in previous studies concerning patients with TAO. In this study, choroidal thickness in patients with TAO decreased by 43.34 µm when axial length increased by 1 mm.

There were some limitations in this cross-sectional study. The contributions of some factors that could affect choroidal thickness, such as the length of previous treatment or the receipt of other medical therapy, were not assessed. Additionally, real-time measurements of retinal and choroidal blood flow were not conducted, which hindered exploration of the mechanisms underlying choroidal thickening.

## Conclusion

SFCT is altered in patients with TAO; it is significantly thicker in patients with active disease. SFCT may be useful as a staging index during disease evaluation. SS-OCT is a non-invasive method for determining the stage of disease; it could be used to evaluate the efficacy of intravenous glucocorticoid therapy.

10.1136/bjo-2023-323694.supp1 Supplementary data

## Data Availability

Data are available on reasonable request.
