# Supplementary material for: Choroidal thickness in patients with thyroid-associated ophthalmopathy, as determined by swept-source optical coherence tomography
Source: Br J Ophthalmol. 2023 Oct 19;108(8):1081–7. doi: 10.1136/bjo-2023-323694 (PMC11287629; doi:10.1136/bjo-2023-323694)
Supplement: Supplementary data [file bjo-2023-323694supp002.pdf]

eTable 1 Characteristics of the patients

	TAO patients n=30	Healthy controls n=38	P value
**Age，y**	45.23±11.92	46.54±18.04	0.9557
**Sex（Female/Male）**	22/8	26/12	0.659
**Duration of disease, y**	2.46±4.39	N/A	
**Treatment history（n，%）**		N/A	
None	12（40%）		
Steroids	15（50%）		
Decompression Surgery	2（6.7%）		
Secukinumab	1（3.3%）		
**Thyroid function（n，%）**		N/A	
Hyperthyroidism	16（53.3%）		
Hypothyroidism	7（23.3%）		
Euthyroidism	7（23.3%）		
**Activity（n，%）**		N/A	
Active phase	19（63.3%）		
Inactive phase	11（36.7%）		
**Severity（n，%）**		N/A	
Moderate to severe	24（80.0%）		
Severe	6（20.0%）		
**Axil Length mm**	24.40±1.58	24.21±1.09	0.5865
**Proptosis mm**	20.28±3.00	14.72±1.50	P＜0.001
**IOP mmHg**	19.51±4.40	16.91±3.97	P＜0.01
**BCVA**	0.77 ±0.29	0.85 ±0.17	0.6187

eFigure 1 a. Comparisons of the SFCT based on SS-OCT image measurements between patients with active ,inactive TAO group and healthy subjects. b. Comparisons of SFCT between mild, moderate to severe, severe TAO group and healthy subjects

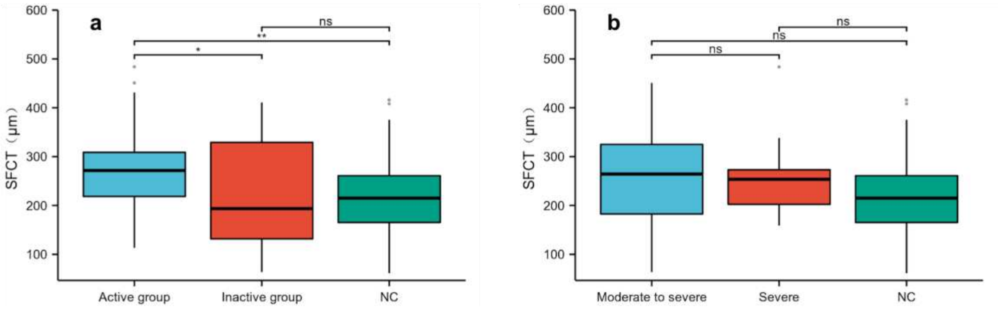

eTable 2 Multivariate linear regression using SFCT as dependent factor in TAO patients

Coefficients[a]

Model		Unstandardized Coefficients		Standardized Coefficients	t	Sig.	Correlations	
		B	Std. Error	Beta			Zero-order	Partial
1	(Constant)	977.346	166.827		5.858	.000		
	Age	-.496	.694	-.061	-.714	.479	.034	-.099
	Duration	.061	2.151	.003	.029	.977	-.374	.004
	Proptosis	1.491	2.743	.045	.544	.589	-.121	.075
	CAS	21.681	8.972	.224	2.417	.019	.531	.318
	BCVA	105.106	35.632	.290	2.950	.005	.115	.379
	IOP	8.277	2.146	.370	3.856	.000	.313	.472
	AL	-43.339	6.022	-.612	-7.197	.000	-.730	-.706
