# Supplementary material for: Choroidal thickness in patients with thyroid-associated ophthalmopathy, as determined by swept-source optical coherence tomography
Source: Br J Ophthalmol. 2023 Oct 19;108(8):1081–7. doi: 10.1136/bjo-2023-323694 (PMC11287629; doi:10.1136/bjo-2023-323694)
Supplement: Supplementary data [file bjo-2023-323694supp001.pdf]

edanz 理文编辑
INNOVATIVE SCIENTIFIC SOLUTIONS

Certificate of Editing

Edited provisional title
Choroidal Thickness in Patients with Thyroid-Associated Ophthalmopathy, as Measured Determined by Swept-Source Optical Coherence Tomography

Client name and institution
Zhong Sisi, Shanghai Jiao Tong University School of Medicine Affiliated Ninth People's Hospital, Ophthalmology.No.639 Zhizaoju Road, Huangpu District, Shanghai, CN. 200011.

Date Completed
2023-06-26

Identification code
602367

Certificate issued by
Koji Yamashita
Managing Director and CEO

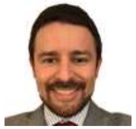

Expert Editor: Ryan Chastain-Gross
2008-BS Microbiology
Ohio State University
Dentistry, Ophthalmology And Optometry, Immunology

www.liwenbianji.cn

While this certificate confirms the authors have used Edanz's editing services, we cannot guarantee that additional changes have not been made after our edits.
